# Implementation of a Potential Industrial Green, Economical, and Safe Strategy to Enhance Commercial Viability of Liquid Self-Nanoemulsifying Drug Delivery System

**DOI:** 10.3390/pharmaceutics17111461

**Published:** 2025-11-12

**Authors:** Abdelrahman Y. Sherif, Mohammad A. Altamimi, Ehab M. Elzayat

**Affiliations:** Department of Pharmaceutics, College of Pharmacy, King Saud University, Riyadh 11451, Saudi Arabia; ashreef@ksu.edu.sa (A.Y.S.); maltamimi@ksu.edu.sa (M.A.A.)

**Keywords:** green pharmaceutical manufacturing, sustainable solidification approach, Box–Behnken, formulation leakage

## Abstract

**Background/Objectives**: Conventional solidification methods for liquid self-nanoemulsifying drug delivery systems face significant limitations. This includes complex manufacturing processes, high costs, and environmental concerns. This study aimed to develop and optimize a thermoresponsive self-nanoemulsifying drug delivery system (T-SNEDDS) for dapagliflozin as a sustainable alternative solidification technique. **Methods**: Oil and surfactant were selected based on solubility and emulsification studies. The Box–Behnken approach was used to examine the impacts of three independent variables (pluronic F127, propylene glycol, and dapagliflozin concentrations) on liquefying temperature and time. Optimized T-SNEDDS was characterized in terms of particle size, zeta potential, and dissolution performance. Stability assessment included centrifugation testing and a six-month storage evaluation. The green pharmaceutical performance was comparatively evaluated against five conventional solidification methods using ten adapted parameters. **Results**: Imwitor 308 and Cremophor EL were selected as optimal excipients for SNEDDS formulation. In addition, Pluronic F127 and propylene glycol were used to induce solidification during storage. The optimized formulation (8.60% *w*/*w* Pluronic F127, 10% *w*/*w* propylene glycol, and 5% *w*/*w* dapagliflozin) exhibited a liquefying temperature of 33.5 °C with a liquefying time of 100.3 s and a particle size of 96.64 nm. T-SNEDDS significantly enhanced dissolution efficiency of dapagliflozin (95.7%) compared to raw drug (42.4%) and marketed formulation (91.3%). Green pharmaceutical evaluation revealed that T-SNEDDS achieved the highest score compared to conventional approaches. **Conclusions**: T-SNEDDS represents a superior sustainable approach for SNEDDS solidification that offers enhancement in drug dissolution while addressing manufacturing, environmental, and economic challenges through its solvent-free and single-step preparation process with excellent scalability potential.

## 1. Introduction

Self-nanoemulsifying drug delivery system (SNEDDS) has been invented to boost the bioavailability of lipophilic drugs [1,2]. Unfortunately, the liquid nature of SNEDDS limits its pharmaceutical applications due to the reported leakage from soft gelatin capsules [3,4]. In this regard, various solidification approaches (fluid-bed coating, spray drying, and lyophilization) have been implemented to overcome this limitation [5]. However, the reported drawbacks, including the need for sophisticated equipment and multiple processing steps, increase both production cycle time and operational costs [5,6]. On the other hand, hot-melt extrusion produces solid SNEDDS with a loss of intrinsic emulsifying tendency and poor content uniformity. Additionally, the susceptibility of drugs to thermal degradation hinders their use for susceptible agents [7]. On the other hand, the adsorption approach faces several challenges, including drug trapping within the carrier agent, increased final dosage volume, and industrial scalability issues [8,9].

In response to demand for an industrially applicable approach with low impact on the environment, thermoresponsive SNEDDS (T-SNEDDS) was implemented in the present study. Generally, the aqueous system containing Pluronic F127 exhibits a transition behavior from liquid to gel state at room and body temperature, respectively. However, the integration of Pluronic F127 within non-aqueous formulations containing propylene glycol as a cosolvent reverses thermoresponsive behavior. In this regard, incorporating both agents within SNEDDS formulation yields a system that remains solid during storage, which undergoes a transition to a liquid state after exposure to body temperature. This reverse behavior enables solid-state storage while ensuring rapid liquefaction and drug release upon oral administration.

The current T-SNEDDS approach presents compelling advantages from manufacturing, environmental, and pharmaceutical perspectives. The simple mixing process eliminates the need for specialized equipment or complex processing steps. The single-step preparation enhances scalability potential and effectively mitigates common scale-up challenges associated with other approaches. Furthermore, complete reversible solidification preserves the original liquid formulation’s intrinsic self-emulsifying properties and ensures the complete release of the loaded agent. Additionally, the solvent-free and energy-efficient process aligns with green pharmaceutical manufacturing principles that address growing sustainability imperatives in the pharmaceutical industry.

**Figure 1:**
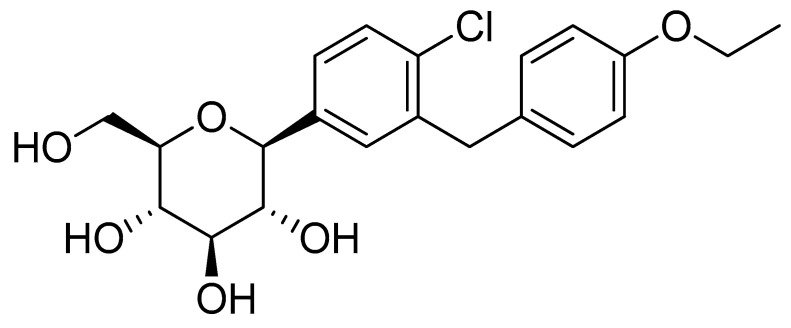
Chemical structure of dapagliflozin.

Dapagliflozin (Dapa, Figure 1) was selected as the model drug in the present study owing to its challenging physicochemical properties. Dapa is a non-ionic compound with the molecular formula C_21_H_25_ClO_6_, a molecular weight of 408.88 g/mol, and has a reported log *p* value of 2.7 [10,11]. Its chemical structure contains two hydrophobic benzene rings, which create unfavorable hydrophobic-hydrophilic interactions with the aqueous media. Consequently, the T-SNEDDS formulation could be used to enhance its dissolution profile. Dapa belongs to a sodium-glucose cotransporter-2 (SGLT2) inhibitor, which increases glucose excretion in the urine [12,13]. This mechanism of action reduces blood glucose levels through an insulin-independent effect [14]. Therefore, it could be used in patients with insulin resistance or impaired pancreatic function [15].

The present study aimed to develop and optimize a T-SNEDDS formulation for dapagliflozin using a Design of Experiments (DoE) approach. The influence of three critical formulation variables (Pluronic F127 concentration, propylene glycol concentration, and dapagliflozin concentration) on liquefying temperature and liquefying time was investigated. After that, the formulation’s physicochemical properties were evaluated, and its dissolution performance was compared to that of the raw drug and commercial formulation. Moreover, the stability of the formulation under rigorous transportation conditions and the potential for leakage were evaluated. Finally, a comprehensive evaluation of green pharmaceutical performance was performed between T-SNEDDS and conventional solidification technologies in terms of environmental impact, cost-effectiveness, and operator safety parameters.

## 2. Materials and Methods

### 2.1. Materials

Riyadh Pharma (Riyadh, Saudi Arabia) kindly donated Dapagliflozin (Dapa). Avonchem (Cheshire, UK) supplied oleic acid (free fatty acid). Sasol Germany GmbH (Hamburg, Germany) and Gattefosse (Saint-Priest, France) provided imwitor-308 (short-chain monoglyceride) and Peceol (long-chain monoglyceride), respectively. Abitec Corporation (Janesville, WI, USA) and John L. Seaton & Co., Ltd., Croda International Plc. (East Yorkshire, UK) Supplied Captex 355 EP/NF (medium-chain triglyceride) and Soybean oil (long-chain triglyceride), respectively. BASF (Ludwigshafen, Germany) and Merck-Schuchardt OHG (Hohenbrunn, Germany) provided Cremophor EL and Tween 60, respectively. Merck (Darmstadt, Germany) and Nikkol Chemical Co. (Tokyo, Japan) supplied Span-80 and TO-10v, respectively.

### 2.2. Quantification Method for Drug Analysis

Dapagliflozin quantification was performed using an Ultimate 3000 UPLC system. Isocratic elution of the mobile phase was attained using a connected quadratic pump through a column (BEH C18) placed in a column chamber with a temperature manager system. Before sample analysis, the column temperature was set to 25 °C, and the organic phase (acetonitrile) and aqueous phase (0.1% formic acid solution) were eluted at a 60:40 (*v*/*v*) ratio with a flow rate of 0.4 mL/min to achieve equilibrium. The connected Photodiode Array (PDA) detector was used to measure drug absorption at 222 nm. A calibration curve was constructed using dapagliflozin standard solutions prepared at concentrations ranging from 0.5 to 20 μg/mL. The method demonstrated excellent linearity with a correlation coefficient (r^2^) of 0.9999.

### 2.3. Dapagliflozin Solubility in Oils

Numerous kinds of oils were chosen to study the solubility of dapagliflozin and to determine the optimal oil phase for the preparation of T-SNEDDS formulations. Using a magnetic stirrer, oil was mixed with an excess amount of dapagliflozin in a 4 mL glass vial at 1000 rpm. The next day, the oil-drug mixture was centrifuged for 30 min at 10,000 rpm to precipitate the undissolved. An Eppendorf tube was first placed on the analytical balance, and the balance was auto-zeroed. After that, one drop from the supernatant (15–26 mg) was added to the Eppendorf using a micropipette, and its weight was recorded. Afterwards, 1.8 mL of acetonitrile was added, and drug extraction was attained after sonication. The obtained acetonitrile solution containing dapagliflozin was diluted appropriately to ensure it fell within the range of the constructed calibration curve, and the drug concentration was estimated using the developed UPLC method.

### 2.4. Emulsification Study

The present study was conducted to select an appropriate surfactant with superior emulsification properties for the chosen oil phase. Different types of surfactants (Cremophor EL, Tween 60, Span 80, and TO-10v) and imwitor 308 were mixed in a one-to-one ratio and placed in an incubator set at 40 °C to form a homogenous mixture. The prepared surfactant oil mixture was diluted in a ratio of 1 to 200 using distilled water to enhance its dispersion. A UV-visible spectrophotometer (UV-1700, Shimadzu, Kyoto, Japan) was used to measure the % transmittance at 638 nm [16].

### 2.5. Thermorepositive Study

Pluronic F127 was selected as a thermoresponsive agent in the present study. Non-aqueous components (ethanol, propanol, propylene glycol, and glycerol) were used to select an optimum cosolvent system for Pluronic F127 within T-SNEDDS. The non-aqueous components were mixed with Pluronic F127 to study its solubility at room temperature. After that, the mixtures were incubated at 40 °C to facilitate their solubilization. Finally, the soluble mixtures were placed in a refrigerator to assess their propensity for solidification.

### 2.6. Design of Experiments

#### 2.6.1. Selection Variables and Their Ranges

T-SNEDDS is composed of oil, surfactant, propylene glycol, Pluronic F127, and dapagliflozin. The last three formulation components were selected as independent variables based on their impact on thermoresponsive behavior. The selected ranges were as follows: Pluronic F127 concentration (5–10% *w*/*w*), propylene glycol concentration (10–30% *w*/*w*), and dapagliflozin concentration (1–5% *w*/*w*). The lower limit of 5% *w*/*w* for Pluronic F127 was selected to ensure adequate solidification at room temperature and the formation of a complex matrix. On the other hand, 10% *w*/*w* propylene glycol was chosen as a lower limit to ensure complete dissolution of Pluronic F127 within the lipid-based matrix. Regarding dapagliflozin, 1% *w*/*w* was selected as the minimum reasonable dosage form size that contains the therapeutic dose (10 mg) of the drug, which can be filled into a single hard gelatin capsule. The upper limit for Pluronic F127 and propylene glycol was set to be 10 and 30%*w*/*w*, respectively. This provides a reasonable range and ensures a complete understanding of their impact on formulation behavior. A 5% *w*/*w* drug loading was selected for dapagliflozin, which provides an acceptable dosage volume (200 mg) of the formulation containing a therapeutic dosage of drug (10 mg).

#### 2.6.2. Experimental Design and Statistical Analysis

Design-Expert^®^ software (version 13, Stat-Ease Inc., Minneapolis, MN, USA) was used to investigate the influence of three selected independent variables on the two measured responses. A Box–Behnken Design was used to achieve this purpose and to select an optimized T-SNEDDS formulation. The chosen design involves three levels, ensuring good predictive capability and efficiently accounting for quadratic influences with fewer experimental runs. The two measured responses were liquefying temperature (°C) and liquefying time (seconds). Table 1 shows the 17 suggested formulations generated by the DoE software. The model was selected based on the sum of squares, lack-of-fit tests, and statistical analysis of R^2^ values (both adjusted and predicted), Adequate precision (signal-to-noise ratio), and ANOVA (*p* < 0.05 was considered significant).

### 2.7. Preparation of SNEDDS Formulation

T-SNEDDS formulations suggested by the Design of Experiments software in Table 1 were prepared using a simple mixing approach. Based on the concentrations provided in Table 1, the amount of propylene glycol, poloxamer, and dapagliflozin was accurately weighed and placed in a 5 mL glass beaker. The concentration of imwitor and Cremophor EL mixture could be estimated by subtracting the sum of other components’ concentrations from 100, while keeping the ratio of imwitor and Cremophor EL at 1:1. Afterward, they were mixed with a magnetic stirrer and incubated for 2 h at 40 °C in an incubator to enhance the solubility of Pluronic F127. One gram of prepared T-SNEDDS formulations in a liquid state was placed in test tubes and allowed to solidify to measure the liquifying temperature and time.

### 2.8. Determination of Liquefying Temperature

The liquefying temperature is the temperature at which T-SNEDDS completely transitions from the solid (hard and opaque) to the liquid state (flowable and transparent). Therefore, this test was performed to determine this critical transition temperature. A water bath set at 28 ± 0.5 °C was used for this purpose. Test tubes containing T-SNEDDS were held using a rack and then placed in a water bath for equilibration. After that, test tubes were removed from the water bath and examined to identify the liquified formulations. To identify the liquifying temperature for all formulations, the temperature was increased by 0.5 ± 0.1 °C, and the formulations were monitored for any signs of liquefaction.

### 2.9. Determination of Liquefying Time

This test was conducted to determine the time required for T-SNEDDS formulation to transition from a solid to a liquid state in vivo. The water bath temperature was set at 37 ± 0.1 °C. The liquefying time was determined by measuring the time between immersion of the test tube in a water bath and the complete conversion of T-SNEDDS to a liquid state.

### 2.10. Particle Size and Zeta Potential Measurement

The prepared optimized T-SNEDDS containing Dapa (50 mg/g) was dispersed in distilled water with a dilution factor of 1:1000 for particle size and zeta potential measurements. The dispersed formulation was placed in a cuvette and a folded capillary cell to measure Particle size and zeta potential, respectively. Sample measurement was performed at 25 °C using the Zetasizer instrument Model ZEN3600, Malvern Instruments Co. (Worcestershire, UK) [17].

### 2.11. In Vitro Dissolution

A dissolution apparatus of Type II (LOGAN Inst. Corp., Somerset, NJ, USA) was used to study the in vitro dissolution profile of Dapa from the raw drug, optimized T-SNEDDS, and marketed tablet. To ensure dose equivalence, 10 mg of dapagliflozin was used for all tested agents. For the raw drug, 10 mg was accurately weighed and placed within a hard gelatin capsule. For the optimized T-SNEDDS formulation (containing 5% *w*/*w* dapagliflozin), 200 mg of the formulation was filled into a hard gelatin capsule. The marketed tablet containing 10 mg dapagliflozin was tested directly. The capsule was surrounded with a sinker to facilitate its immersion and avoid floating during the experiment. An equivalent amount of the dissolution medium (900 mL), consisting of phosphate buffer (pH 6.8), was placed in each vessel and heated to 37 ± 0.5 °C before the experiment. At the beginning of the experiment, the test agent was placed in the vessel, and the paddle was allowed to rotate at 50 rpm. Samples were withdrawn from the media at predetermined time intervals (5, 10, 15, 30, 45, and 60 min) using a syringe equipped with a 10-micron filter.

### 2.12. Intrinsic Thermoresponsive Assessment

The current study was performed to investigate the ability of T-SNEDDS to liquefy and re-solidify when exposed to high and low temperatures, respectively. The optimized T-SNEDDS was placed in a glass vial and subjected to five heating and cooling cycles when placed in an incubator set at 37 °C and a refrigerator set at 4 °C, respectively. Images for the T-SNEDDS were captured to visualize physical appearance after exposure to these conditions.

### 2.13. Stability Study

A stability study was conducted to evaluate the performance of T-SNEDDS under vigorous transportation conditions and assess the potential for leakage. For transportation assessment, T-SNEDDS was placed in a 2 mL Eppendorf tube and centrifuged at 5000 rpm for 30 min. After that, the formulation was examined for any sign of phase separation. For leakage assessment, T-SNEDDS was filled into a hard gelatin capsule, which provides a good model for assessing liquefaction tendency. The prepared capsules were stored for six months at room temperature to check for any signs of leakage.

### 2.14. Green Pharmaceutical Performance

A comparative evaluation of green pharmaceutical performance was conducted between T-SNEDDS and conventional SNEDDS solidification methods, including spray drying, lyophilization (freeze-drying), hot-melt extrusion, fluid-bed coating, and adsorption onto solid carriers. Currently, there is no standardized green assessment framework specifically designed for pharmaceutical formulation preparation. Therefore, the 12 Principles of Green Chemistry were used as a framework and modified to align with the current pharmaceutical approach [18]. Ten key parameters were adapted across three primary categories: environmental impact (energy processing, solvent emissions, and waste streams), cost effectiveness (manufacturing complexity, material loss, processing time, and scalability), and operator safety (excipient safety, processing pressure, and washing process). Environmental impact assessments were conducted to align with green chemistry guidelines, while cost evaluations utilized standard economic indicators for pharmaceutical manufacturing. Safety assessments were performed in accordance with occupational health and safety protocols. Each parameter was assigned a three-tier color-coded score based on established green chemistry principles. The scoring criteria were systematically defined as follows: Score 1 (red—high impact), Score 2 (yellow—moderate impact), and Score 3 (green—low impact). Table 2 presents the detailed scoring criteria for all 10 parameters.

## 3. Results and Discussion

The leakage potential of liquid SNEDDS from the capsule limits its commercial viability as a marketed dosage form. Consequently, solidification strategies were implemented to eradicate the leakage and enhance its pharmaceutical applicability. The present study aimed to develop T-SNEDDS as a green, economical, and safe alternative to conventional approaches. To achieve this objective, the current research was divided into four separate phases as follows: selection of T-SNEDDS components, study of the impact of formulation variables on thermoresponsive behavior, pharmaceutical assessment of the optimized formulation, and evaluation of the green pharmaceutical performance.

### 3.1. Selection of T-SNEDDS Components

Liquid SNEDDS are usually composed of oil, surfactant, and cosolvent. The oil was selected based on a measured solubility study, while the surfactant was selected based on transmittance measurement. Herein, a thermoresponsive polymer (Pluronic F127) was chosen to prepare T-SNEDDS, and propylene glycol was selected as a cosolvent to ensure the solubilization of Pluronic F127 in the formulation. The systematic selection for each component was presented in this section to ensure the formulation of T-SNEDDS with the intended thermoresponsive performance.

#### 3.1.1. Oil Selection

The oil phase in the T-SNEDDS formulation was selected based on the measured solubility of dapagliflozin. This enhances drug solubilization within the GIT and provides a concentration gradient driving force to boost its bioavailability [19,20]. Five types of oils were selected based on their fatty acid chain lengths and varying degrees of esterification. Oleic acid consists of free fatty acids, while imwitor 308 and peceol are made up of short-chain and long-chain monoglycerides, respectively. Moreover, captex 355 and soybean oil are selected to represent medium-chain and long-chain triglycerides, respectively. Table 3 shows that triglycerides (captex 355 and soybean oil) had lower drug solubility, with values of 1.71 ± 0.07 and 0.96 ± 0.15 mg/g, respectively. Dapagliflozin solubility increased to 6.91 ± 0.24 mg/g in free fatty acids (Oleic acid). Finally, maximum solubility was attained in monoglycerides (Peceol and imwitor 308) with a value of 67.56 ± 2.64 and 212.48 ± 2.92 mg/g, respectively.

The observed superior solubility of Dapa in oil composed of free fatty acid over triglycerides could be ascribed to its ability to form hydrogen bonds with the free carboxylic groups of oleic acid. This agrees with a previous study that showed dapagliflozin can form hydrogen bond [21]. The superior solubilization capacity of monoglycerides compared to triglycerides can be attributed to their structural differences. Monoglycerides possess free hydroxyl groups on the glycerol backbone that can form hydrogen bonds with dapagliflozin. This facilitates drug-lipid interactions and solubilization of dapagliflozin. In contrast, triglycerides have all hydroxyl groups esterified with fatty acids. This eliminates hydrogen bonding sites and creates greater steric hindrance due to the presence of three bulky fatty acid chains. Furthermore, the measured remarkable drug solubility in monoglycerides could be ascribed to the additional reported emulsification properties [22]. Additionally, present results revealed that decreasing the chain length of fatty acid chains attached to glycerol in mono and triglycerides increased dapagliflozin solubility. Finally, imwitor 308 was chosen as the oil phase to prepare T-SNEDDS owing to the incredible solubilization power.

#### 3.1.2. Surfactant Selection

**Figure 2:**
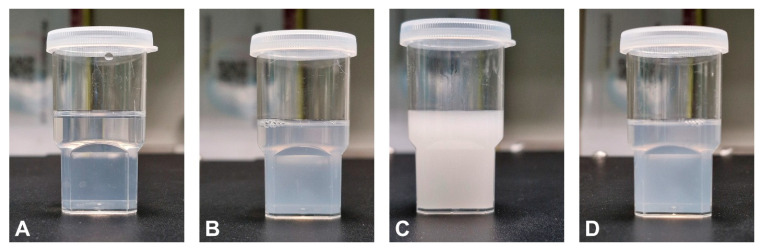
Physical appearance of the dispersed surfactant—imwitor 308 mixtures containing (**A**) Cremophor EL, (**B**) Tween 60, (**C**) Span 80, and (**D**) TO-10v as surfactants.

The surfactant was selected solely based on its emulsification power to ensure the formation of a nanosized emulsion system following dispersion. This is ascribed to the ability of the oil component to achieve desired drug loading. To achieve this, various types of surfactants were mixed with the selected oil phase (imwitor 308) to select the optimum one during formulation preparation. Figure 2 shows the physical appearance of the dispersed surfactant-oil mixtures. Moreover, the transmittance percentage for each surfactant-oil mixture was calculated to give numerical values and presented in Table 4. The present results revealed that Cremophor EL has a high ability to form a nanosized dispersion system, indicated by its clear physical appearance and high transmittance value [23]. Therefore, T-SNEDDS formulations were prepared using Cremophor EL and imwitor 308 as surfactant and oil phase, respectively.

#### 3.1.3. Selection of Solidifying Agents

Pluronic F127 was selected as a thermo-modulating polymer to facilitate the liquefaction and solidification of the SNEDDS formulation. However, Pluronic F127 polymer failed to dissolve in Cremophor EL and imwitor 308. Therefore, the cosolvent component must be added to ensure solubilization of Pluronic F127 within the formulation. Various types of cosolvents (ethanol, propanol, propylene glycol, and glycerol) were selected to achieve this purpose. All cosolvents failed to dissolve the Pluronic F127 polymer when mixed at room temperature. Therefore, they were incubated at 40 °C, and cosolvents (ethanol, propanol, and propylene glycol) were able to dissolve Pluronic F127. However, glycerol was excluded because it failed to achieve this purpose. The obtained solubilized mixtures were placed in a refrigerator to investigate the ability to form a matrix upon cooling. Propylene glycol is the only cosolvent capable of creating this solid matrix. In contrast, the mixture containing ethanol and propanol failed to form this matrix, resulting in the precipitation of Pluronic F127. Therefore, Pluronic F127 and propylene glycol were selected to induce solidification of T-SNEDDS during storage.

**Figure 3:**
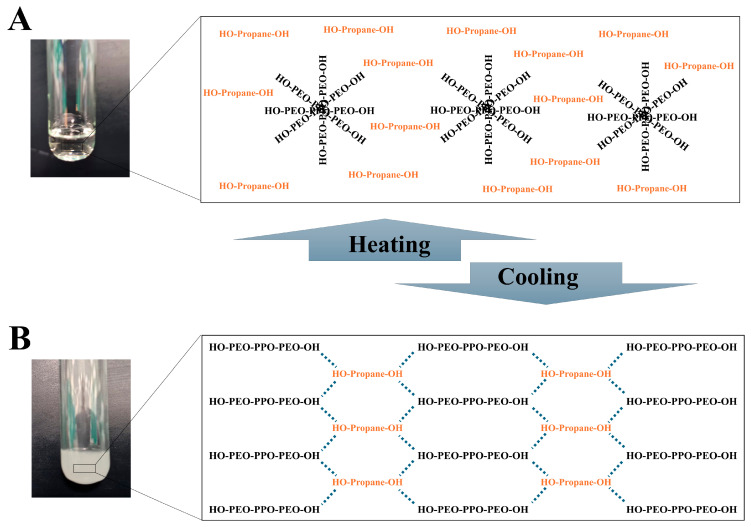
Physical appearance and anticipated mechanism for phase transition of (**A**) liquefied and (**B**) solidified T-SNEDDS after exposure to heating and cooling, respectively.

The Pluronic F127 polymer exhibited a transition to a liquid state at a lower temperature, while it converted to a solid matrix when exposed to higher temperatures [24]. However, the non-aqueous nature of T-SNEDDS could be responsible for the observed inverse thermoresponsive behavior. Figure 3 shows the expected thermoresponsive behavior of Pluronic F127 within T-SNEDDS containing propylene glycol. It is anticipated that monomeric units of Pluronic F127 are arranged in a micellar structure at high temperatures (Figure 3A) [25]. Therefore, once the T-SNEDDS is heated to a temperature above its liquefying temperature, the terminal hydroxyl groups of pluronic are oriented towards the outer surface of the formed micellar structure. Consequently, they could form hydrogen bonding with the hydroxyl groups of propylene glycol, and T-SNEDDS could become liquid. On the other hand, at low temperatures, Pluronic polymers exist as discrete monomers [26]. Monomers of Pluronic F127 could form hydrogen bonds between their terminal hydroxyl groups or with propylene glycol, as presented in Figure 3B [27]. Therefore, the T-SNEDDS is converted to a solid state when it is exposed to low temperatures.

### 3.2. Study the Effect of Formulation Factors on Thermoresponsive Behavior

#### 3.2.1. Model Selection

Three independent factors (pluronic F127 concentration, propylene glycol concentration, and dapagliflozin concentration) were selected to study their influence on the measured responses (liquifying temperature and liquifying time) using DoE software. Seventeen suggested formulations were prepared, and the actual measured response values are presented in Table 5.

**Figure 4:**
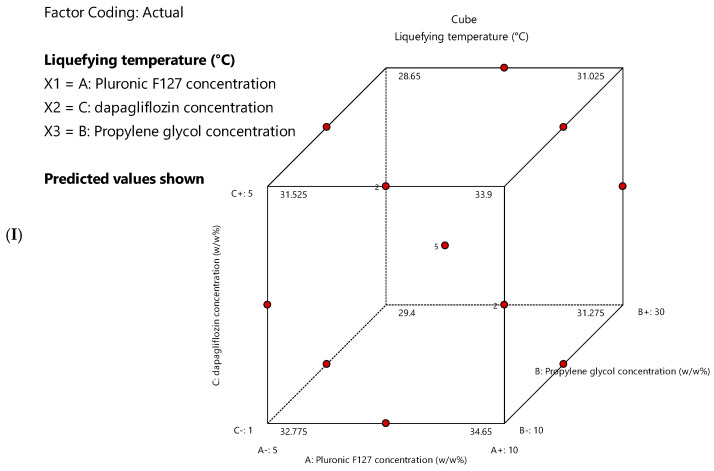

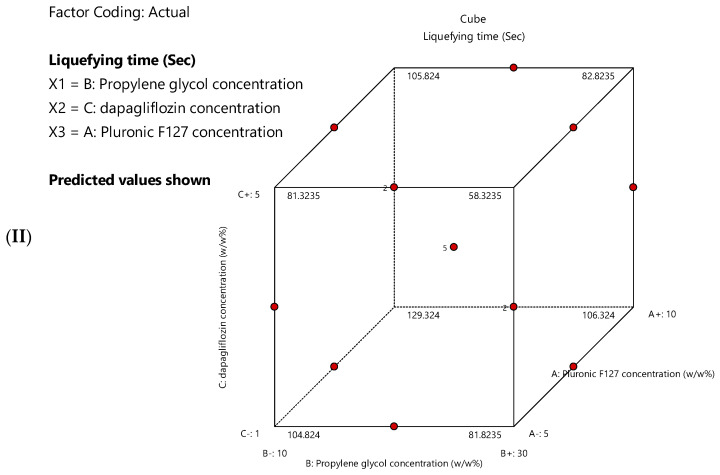
Cube plots show the predicted values of (**I**) liquefying temperature and (**II**) liquefying time at the corners of the experimental design space.

Various mathematical models (linear, two-factor interaction, cubic, and quadratic) were examined by DoE software to study the influence of independent factors on the measured response discretely. The selected model for each response is presented in Table 6, based on the ANOVA analysis performed using the DoE software. The quadratic and linear models were selected by DoE software for liquifying temperature and time, respectively. The selected models were significant, with a non-significant lack of fit, and the estimated R^2^ (both adjusted and predicted) differed by less than 0.2. Figure 4 shows cube plots displaying the values of liquefying temperature and liquefying time at the corners of the experimental design space. The plots demonstrate how the responses vary across different combinations of the three independent variables.

#### 3.2.2. Effect of Liquefying Temperature

**Figure 5:**
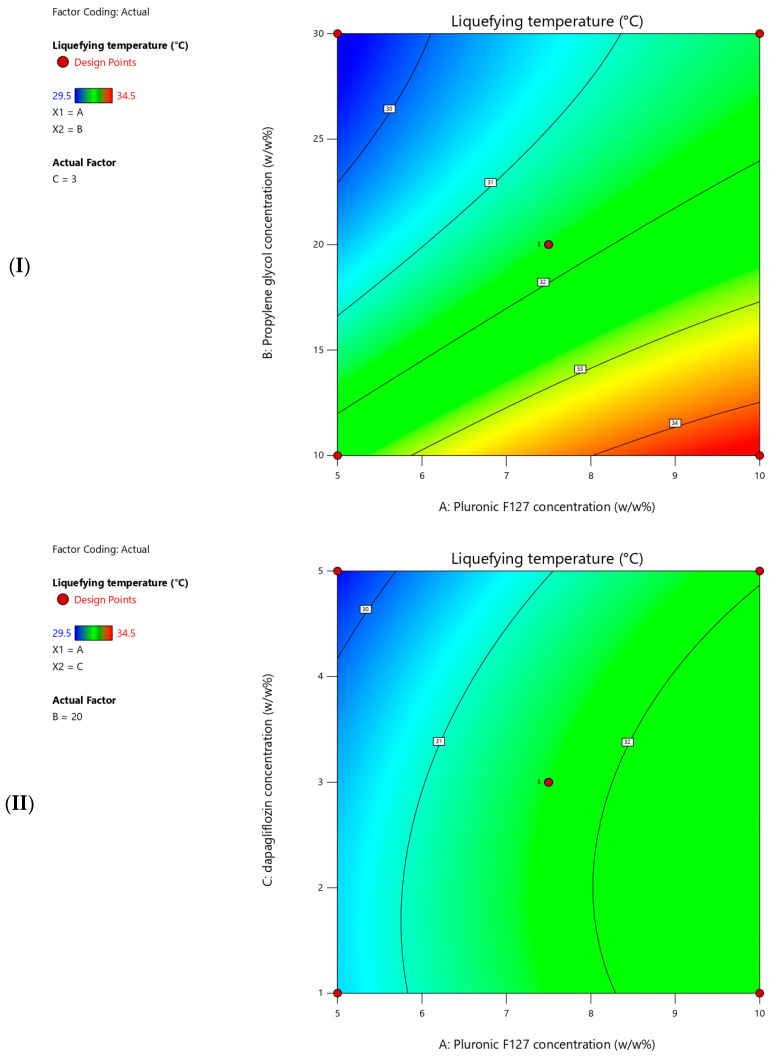

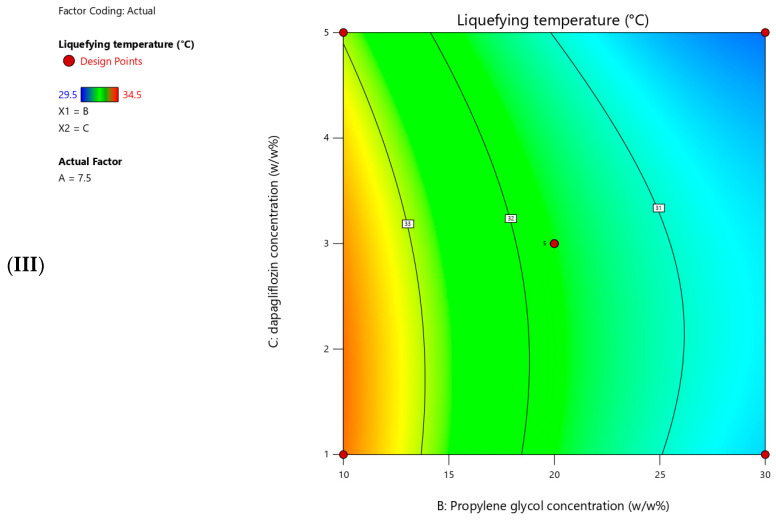
Contour plots showing the effects of (**I**) pluronic F127 (5–10% *w*/*w*) and propylene glycol (10–30% *w*/*w*) concentrations, (**II**) pluronic F127 (5–10% *w*/*w*) and dapagliflozin (1–5% *w*/*w*) concentrations, (**III**) propylene glycol (10–30% *w*/*w*) and dapagliflozin (1–5% *w*/*w*) concentrations on liquefying temperature (°C). The third variable was kept constant at the center point value.

The measured liquefying temperatures for the prepared T-SNEDDS ranged from 29.5 to 34.5 °C (Table 5). Figure 5 shows contour plots for the measured liquefying temperatures. Moreover, Table 7 summarizes the statistical analysis for the significant impact of each factor on measured responses. Additionally, a coded equation (Equation (1)) revealed a second-order polynomial model. This could be used to predict the directional impact degree of independent factors, the interaction between two variables, and the non-linear influence of their interaction on the liquefying temperature. The present results revealed that increasing the Pluronic F127 concentration significantly increased the liquefying temperature of T-SNEDDS (Table 6 and Equation (1)). On the contrary, increasing the propylene glycol and dapagliflozin concentrations significantly reduces the liquefying temperature of T-SNEDDS (Table 6 and Equation (1)). Propylene glycol had the strongest effect on liquefying temperature indicated by large coefficient factor value (1.56), followed by pluronic F127 (1.06). However, dapagliflozin showed minor influence, indicated by the smallest coefficient factor value (0.38). Moreover, the sign of interaction terms between each pair of variables represents synergistic or antagonistic combined effects between them. Finally, the numerical coefficient values of the quadratic terms indicate both the magnitude and direction of each factor’s influence on liquefying temperature.

Liquefying temperature = 31.70 + 1.06 × Pluronic F127 concentration − 1.56 × Propylene glycol concentration − 0.38 × Dapagliflozin concentration + 0.13 × Pluronic F127 concentration × Dapagliflozin concentration + 0.13 × Propylene glycol concentration × Dapagliflozin concentration − 0.23 × Pluronic F127 concentration^2^ + 0.53 × Propylene glycol concentration^2^ − 0.35 × Dapagliflozin concentration^2^ (1)

The liquefying temperature was selected as the response to ensure that T-SNEDDS remains in a solid state during storage and to ensure complete liquefaction at body temperature (37 °C) for optimal in vivo performance. The observed increment in liquefying temperature while increasing pluronic F127 concentration could be ascribed to the formation of a highly cross-linked rigid matrix. This postulation agreed with previous studies showing that increasing polymer concentration increased intermolecular bonding [28]. Therefore, high temperatures are required to break these bonds and convert polymer monomers into micellar structures. On the other hand, increasing the concentration of propylene glycol and dapagliflozin could interfere with the formation of a rigid matrix and decrease the liquefying temperature. For example, Alruwailia et al. reported that dapagliflozin could form a hydrophobic interaction with pluronic polymer units [29]. Similarly, Liapunov et al. reported that propylene glycol forms a hydrogen bond with pluronic [30]. It could be concluded that increasing both agents reduces the intermolecular interaction between long chains of pluronic molecules and reduces the strength of the formed matrix. Consequently, low temperatures are required to break bonds between pluronic F127 monomers in formulations with higher propylene glycol and dapagliflozin concentrations.

#### 3.2.3. Liquefying Time

**Figure 6:**
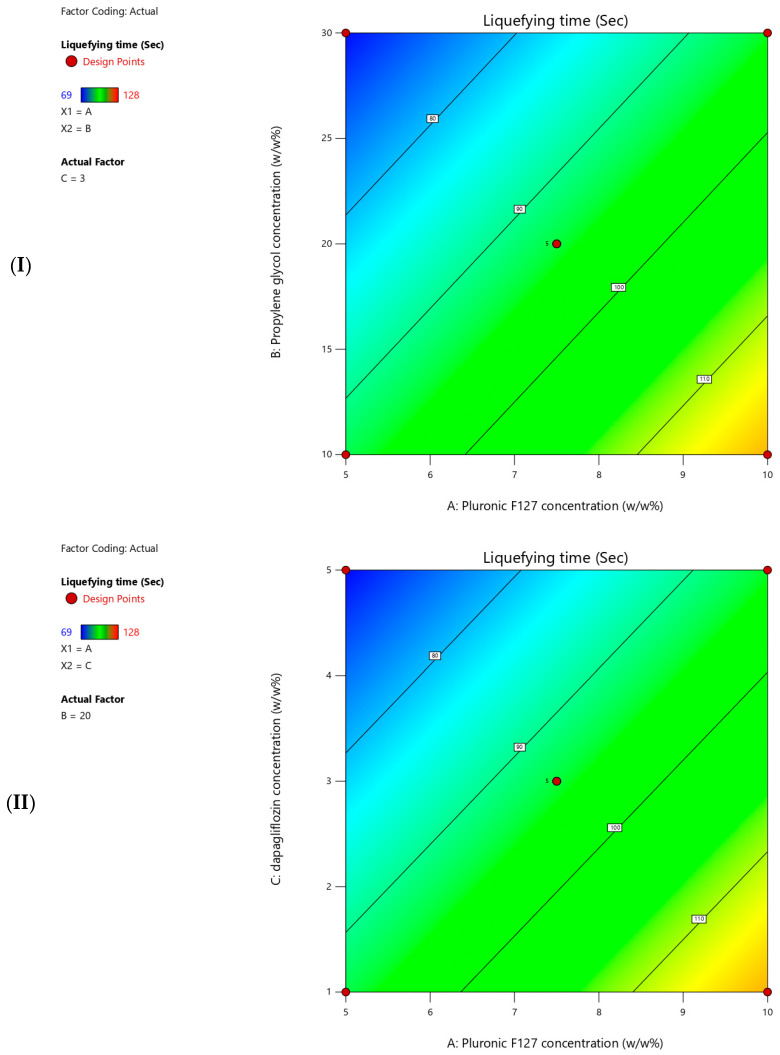

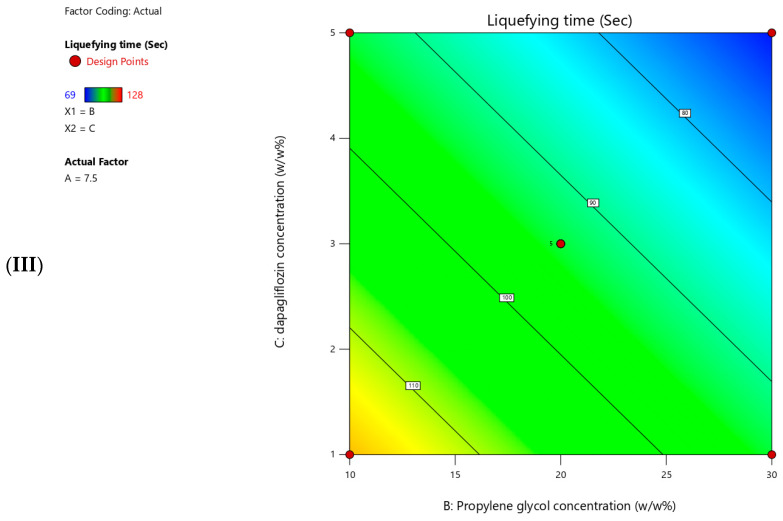
Contour plots showing the effects of (**I**) pluronic F127 (5–10% *w*/*w*) and propylene glycol (10–30% *w*/*w*) concentrations, (**II**) pluronic F127 (5–10% *w*/*w*) and dapagliflozin (1–5% *w*/*w*) concentrations, (**III**) propylene glycol (10–30% *w*/*w*) and dapagliflozin (1–5% *w*/*w*) concentrations on liquefying time (seconds). The third variable was kept constant at the center point value.

The measured liquefying time for the prepared T-SNEDDS ranged from 69 to 128 s (Table 5). Figure 6 shows contour plots for the measured liquefying time. Moreover, Table 7 summarizes the statistical analysis for the significant impact of each factor on measured responses. In addition, a coded equation (Equation (2)) could be used to predict the degree of impact of independent factors and their directional influence on the liquefying time. The present results revealed that increasing the Pluronic F127 concentration significantly increased the liquefying time of T-SNEDDS (Table 6 and Equation (2)). On the contrary, increasing the propylene glycol and dapagliflozin concentrations significantly reduced the liquefying time of T-SNEDDS (Table 6 and Equation (2)).

Liquefying time = 93.82 + 12.25 × Pluronic F127 concentration − 11.50 × Propylene glycol concentration − 11.75 × Dapagliflozin concentration(2)

The current response was measured to select an optimized T-SNEDDS formulation with rapid liquefying performance to ensure a fast clinical outcome in vivo. The observed increment in liquefying time while increasing pluronic concentration could be ascribed to the formation of extensive intermolecular bonds and resulted in the formation of a rigid matrix. Therefore, more time is required to break these bonds and convert polymer monomers into micellar structures. On the other hand, the incorporation of small molecules of propylene glycol and dapagliflozin concentration could interfere with the formation of a rigid matrix and decrease liquefying time. Consequently, less time is required to break bonds between pluronic F127 monomers in formulation with higher propylene glycol and dapagliflozin concentrations. The present finding aligns with the fundamental principles of polymer network formation, whereas the relative size of the used agent could determine the overall matrix strength and its breakdown kinetics [31,32].

#### 3.2.4. Selection of Optimized T-SNEDDS

**Figure 7:**
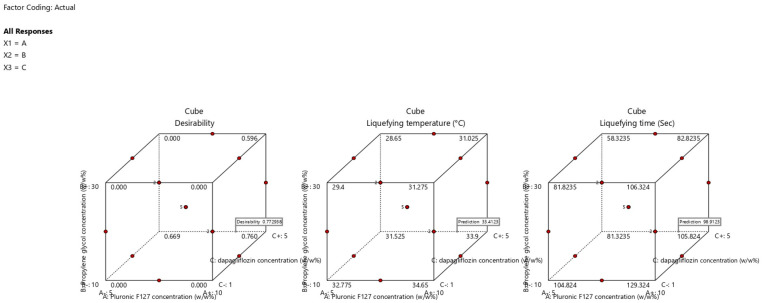
Cube plots display the optimization results for desirability (0.773), liquefying temperature (33.4 °C), and liquefying time (98.9 s) based on optimization criteria.

The optimization of the T-SNEDDS formulation was attained using DoE software based on the following criteria. Increasing the dapagliflozin concentration, which increases drug loading and reduces the total dosage of the formulation. In addition, maximizing the liquefying temperature to prevent premature liquification during storage. Finally, minimizing liquefying time ensures rapid T-SNEDDS conversion to the liquid state in vivo after oral administration. The suggested optimized formulation consisted of 8.60% *w*/*w* Pluronic F127, 10% *w*/*w* propylene glycol, and 5% *w*/*w* dapagliflozin. Figure 7 shows that DoE suggests a T-SNEDDS formulation with remarkable desirability based on the selected criteria.

The suggested T-SNEDDS was prepared, and the predicted and actual values of measured responses are presented in Table 8. The current results indicate that the data mean values fall within the 95% prediction interval suggested by the DoE software. These results demonstrate the accuracy and reliability of selected models and their ability to predict the actual values of responses within the selected range. The prepared optimized T-SNEDDS formulation was exposed to physicochemical characterization, in vitro dissolution, and stability study.

### 3.3. Characterization of Optimized T-SNEDDS

#### 3.3.1. Particle Size and Zeta Potential Measurement

**Figure 8:**
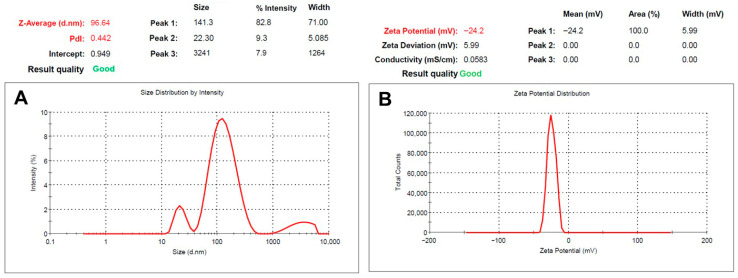
(**A**) particle size and (**B**) zeta potential chromatograms for dispersed optimized T-SNEDDS containing Dapa (50 mg/g).

The particle size distribution and zeta potential for the dispersed nanoemulsion are presented in Figure 8. The results showed that optimized T-SNEDDS produce nanoemulsion particles with a Z-average diameter of 96.64 nm, as determined by cumulant analysis. However, a distribution analysis revealed a predominant peak at 141.3 nm (82.8%), with minimal populations at 22.3 nm (9.3%) and 3241 nm (7.9%). Therefore, the predominant presence of dispersed nanoemulsion particles within the nano size range (>90%) is anticipated to enhance the oral bioavailability of dapagliflozin based on previously reported studies. For example, it has been reported that the SNEDDS formulation significantly increased the bioavailability of the loaded drug by increasing the surface area provided by the dispersed nanoemulsion following oral administration [33]. In addition, the incorporation of loaded drugs into the dispersed nanoemulsion enhanced intestinal permeation via transcellular and paracellular pathways [34]. The measured zeta potential of −24.2 mV (Figure 8B) indicates electrostatic stabilization of the nanoemulsion particles following aqueous dispersion. Furthermore, the negative surface charge can positively influence interactions and migration through the mucin layer covering the intestinal membrane. This could enhance the drug’s absorption within the nanoparticles’ cores following cellular uptake.

#### 3.3.2. In Vitro Dissolution

**Figure 9:**
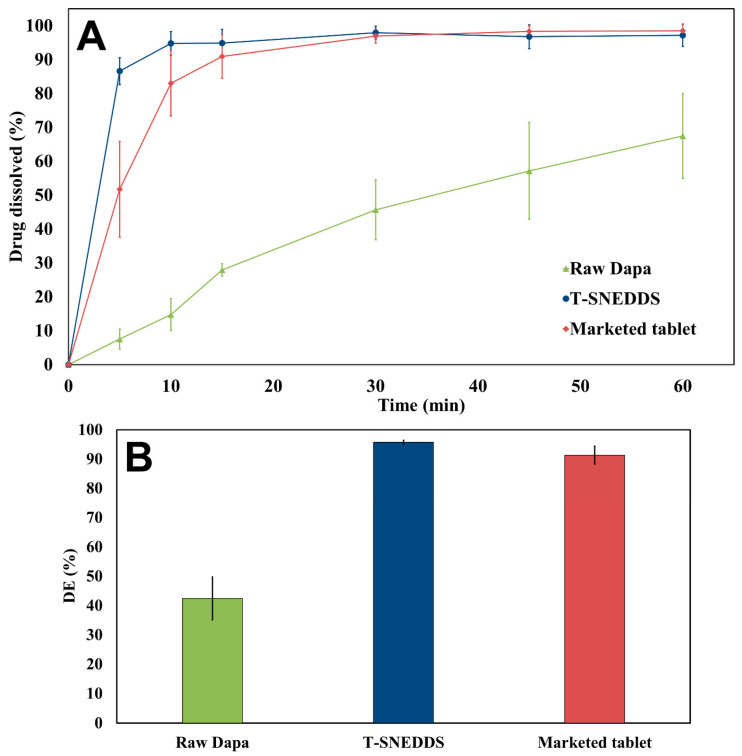
(**A**) In vitro dissolution profiles and (**B**) dissolution efficiency of raw Dapa, T-SNEDDS, and marketed tablet.

Figure 9 presents the in vitro dissolution profile of raw Dapa, optimized T-SNEDDS, and the marketed tablet. This experiment was performed to study the ability of T-SNEDDS to enhance drug dissolution and compare it with the marketed tablet. The results clearly show that T-SNEDDS increased the dissolution efficiency of Dapa from 42.4% to 95.7% compared with the raw drug. Moreover, T-SNEDDS was able to enhance the dissolution efficiency of Dapa compared with a marketed tablet from 91.3 to 95.7%. The complete dissolution of the drug at the end of the experiment confirms its solubilization within the core of the dispersed nanoemulsion. This provides indirect evidence against the occurrence of drug precipitation following its solubilization from the media. The prepared T-SNEDDS formulation is anticipated to augment Dapa bioavailability based on the current dissolution data.

#### 3.3.3. Intrinsic Thermoresponsive Assessment

**Figure 10:**
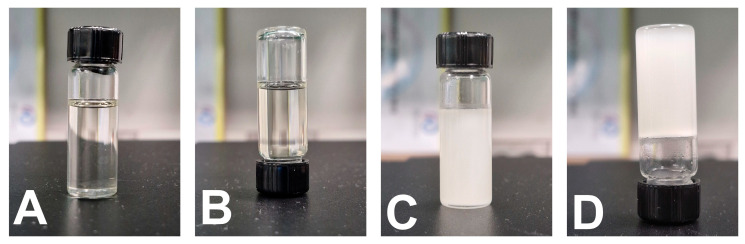
Physical appearance of optimized T-SNEDDS formulation. (**A**,**B**) liquid state after exposure to body temperature before and after flipping, respectively. (**C**,**D**) solidified T-SNEDDS before and after flipping, respectively.

The prepared optimized T-SNEDDS were able to liquefy and re-solidify following exposure to cooling and heating cycles. Figure 10A,B show the physical appearance of the liquid SNEDDS, which exhibits free-flowing properties after flipping. However, Figure 10C,D show that flipping solidified T-SNEDDS formulations with no flowing tendency. This reveals the ability of the T-SNEDDS formulation to maintain its thermoresponsive performance following exposure to cooling and heating cycles.

#### 3.3.4. Stability Study

**Figure 11:**
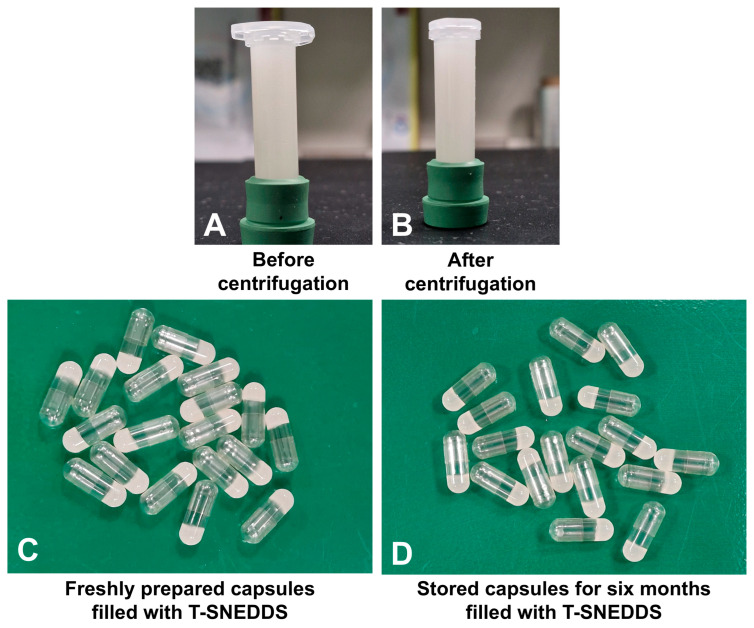
Physical appearance of optimized T-SNEDDS formulation. (**A**) before centrifugation and (**B**) after centrifugation at 5000 rpm for 30 min. (**C**) Freshly prepared hard gelatin capsules filled with T-SNEDDS and (**D**) after six months of storage at room temperature.

Figure 11A,B show the physical appearance of optimized T-SNEDDS before and after exposure to centrifugation, respectively. The T-SNEDDS was capable of tolerating centrifugation forces without phase separation or change in physical appearance. This indicates the ability of SNEDDS formulation to withstand vigorous transportation conditions. Moreover, Figure 11C shows the physical appearance of freshly filled hard gelatin capsules containing T-SNEDDS, while Figure 11D shows their appearance after six months of storage. The absence of formulation leakage and retention of the solid state proves that the current approach successfully resolves the reported leakage issue associated with liquid SNEDDS.

### 3.4. Green Pharmaceutical Performance

Table 9 presents a comprehensive evaluation of the most widely used solidification approaches compared to T-SNEDDS across all ten parameters.

#### 3.4.1. Environmental Impact

Energy processing was assessed based on the energy requirements for instruments, including heating and cooling systems, and vacuum systems. Lyophilization received the lowest score (Score 1) due to the combination of prolonged refrigeration system operation and the requirements of the vacuum pump. Spray drying and fluid bed coating achieved a moderate score (Score 2) as they require a vacuum during the atomization and drying process, respectively. Finally, hot melt extrusion, adsorption, and T-SNEDDS achieved a score of 3 owing to the elimination of the need for a heating/cooling system and vacuum requirements.

Solvent emissions were evaluated based on the type of solvents used throughout the solidification process. Spray drying received the lowest score (Score 1) due to extensive reliance on volatile organic solvents. Lyophilization and fluid bed coating achieved a moderate score (Score 2) as they may require minimal organic solvents. Hot melt extrusion, adsorption, and T-SNEDDS achieved the highest score (Score 3) as they are inherently solvent-free processes.

Waste streams assessment considered the amount and nature of waste generated during the solidification process. Spray drying received a score of 1 due to the extensive waste generated from solvent use and the production of volatile products during processing. However, lyophilization and fluid bed coating scored 2 points due to the demand for a solvent collector and a specialized air-connected system, respectively. Hot melt extrusion, adsorption, and T-SNEDDS achieved a Score of 3 owing to the reduction in waste generation from the used solvents.

#### 3.4.2. Cost Effectiveness

Manufacturing complexity was assessed based on the demand for complex equipment requirements and post-processing needs. Lyophilization and spray drying received the lowest score (Score 1) due to the combination of complex vacuum systems and mandatory post-processing steps, including grinding and sieving. Fluid bed coating and hot melt extrusion achieved a moderate score (Score 2) as they require vacuum systems and demand post-processing of the final product, respectively. Adsorption and T-SNEDDS achieved the highest score (Score 3) by eliminating the demand for a vacuum system and further processing steps.

Material loss was evaluated based on the product’s physical characteristics and the number of steps that could potentially result in material loss. Lyophilization, spray drying, and hot melt extrusion received a Score of 1, as they produce fluffy, low-density powders that are prone to dispersion through multiple processing steps. Fluid bed coating and adsorption scored 2 due to the multiple handling steps and the fluffy nature of the products, respectively. However, T-SNEDDS achieved the highest score (Score 3) due to its liquid nature. This eliminates flying and dispersion issues associated with powder handling.

Processing time directly impacts the cost of the final product and manufacturing throughput. Lyophilization received a Score of 1 due to extended freeze-drying cycles exceeding 48 h plus additional post-processing time. Hot melt extrusion, spray drying, and fluid bed coating scored 2 owing to the demand for post-processing steps. Adsorption and T-SNEDDS achieved a Score of 3 due to their direct filling within capsules without any further processing.

Scalability determines whether laboratory formulations can be successfully scaled up for commercial production. Adsorption and lyophilization received a score of 1 due to challenges in maintaining uniform conditions at larger scales. Fluid bed coating scored 2 as chamber size variations affect product uniformity. Hot melt extrusion and spray drying achieved a Score of 3 due to the straightforward equipment enlargement required for scale-up. Similarly, T-SNEDDS received a Score of 3 due to its simple production method, which facilitates predictable scale-up.

#### 3.4.3. Operator Safety

Excipient safety focuses on solvent toxicity and the associated risks of operator exposure. Spray drying also received a score of 1 due to the use of volatile organic solvents. Lyophilization received a Score of 2 due to the potential use of organic solvents. Fluid bed coating achieved a Score of 3 by utilizing aqueous systems. Likewise, hot melt extrusion, adsorption, and T-SNEDDS achieved a Score of 3, as they use solvent-free approaches.

Processing pressure was evaluated based on the requirements for either a vacuum or a high-pressure system. Spray drying received a Score of 1 due to the combination of vacuum requirements and organic solvent usage, which increases the chance of expulsion. Lyophilization and fluid bed coating scored 2 owing to the inclusion of a vacuum system with aqueous systems. Hot melt extrusion, adsorption, and T-SNEDDS scored 3 as they operate at ambient atmospheric pressure.

Equipment cleaning between batches is critical to prevent cross-contamination. Spray drying and fluid bed coating received a Score of 1 due to extensive cleaning requirements for nozzles and multiple chambers. Lyophilization and hot melt extrusion scored 2 points due to the requirement for moderate cleaning of large parts or disassembly of components. Adsorption and T-SNEDDS achieved a Score of 3 utilizing simple equipment that is easy to clean.

#### 3.4.4. Overall Assessment

T-SNEDDS demonstrated superior overall performance compared to conventional solidification methods. It achieves the highest green pharmaceutical score compared to the other methods. This exceptional performance arises from its solvent-free, single-step manufacturing process, which operates at atmospheric pressure with minimal energy consumption. The liquid nature prevents material loss, while the complete reversibility of solidification preserves intrinsic self-emulsifying properties. T-SNEDDS successfully addresses critical pharmaceutical manufacturing challenges across environmental sustainability, cost-effectiveness, and operator safety.

## 4. Conclusions

This study successfully demonstrates that thermoresponsive self-nanoemulsifying drug delivery system (T-SNEDDS) represents a promising alternative in pharmaceutical manufacturing. T-SNEDDS solves the leakage problem associated with liquid SNEDDS through a temperature-dependent solidification approach. It remains solid during storage at 25 °C and rapidly liquefies at body temperature (37 °C). The optimized formulation consisted of 8.60% *w*/*w* Pluronic F127, 10% *w*/*w* propylene glycol, and 5% *w*/*w* dapagliflozin. It exhibited a liquefying temperature of 33.5 °C and a liquefying time of 100.3 s. The dispersed optimized T-SNEDDS produces a nanoscale emulsion with a particle size of 96.64 nm. Moreover, it successfully enhanced the dissolution of dapagliflozin, achieving 95.7% dissolution efficiency compared to 42.4% for the raw drug. Furthermore, stability studies revealed the ability to maintain the integrity of the solid state without any distortion during six-month storage. T-SNEDDS overcomes the manufacturing and economic barriers associated with conventional solidification methods through a single-step mixing process. This eliminates the need for sophisticated equipment, complex multi-step processes, and high energy consumption. Additionally, it addresses environmental sustainability concerns through its solvent-free, energy-efficient process, which operates at atmospheric pressure with minimal waste generation. This study represents a promising proof-of-concept that warrants future investigation through pilot-scale and industrial-scale validation studies to confirm these theoretical advantages in manufacturing settings.

## Figures and Tables

**Table 1:** Suggested formulation by Design of Experiments.

STD	Run	Pluronic F127 Concentration (Thermoresponsive Polymer)	Propylene Glycol Concentration (Cosolvent)	Dapagliflozin Concentration (Therapeutic Drug)
3	1	5	30	3
1	2	5	10	3
5	3	5	20	1
9	4	7.5	10	1
16	5	7.5	20	3
14	6	7.5	20	3
4	7	10	30	3
10	8	7.5	30	1
6	9	10	20	1
15	10	7.5	20	3
8	11	10	20	5
13	12	7.5	20	3
12	13	7.5	30	5
17	14	7.5	20	3
7	15	5	20	5
11	16	7.5	10	5
2	17	10	10	3

The concentration in the table is expressed as a percentage by weight (*w*/*w*). Imwitor (oil) and Cremophor EL (surfactant) ratio was kept at 1:1 in all formulations. The concentration of imwitor and Cremophor EL mixture could be estimated by subtracting the sum of other components from 100.

**Table 2:** Scoring criteria for green pharmaceutical performance assessment.

Parameter	Score 1 (Red)	Score 2 (Yellow)	Score 3 (Green)
Energy Consumption	Vacuum and extensive heating (>150 °C) or cooling (<−50 °C)	Vacuum or extensive heating/cooling	No need for an intensive energy consumption method
Solvent Usage	Organic solvents required	Minimal organic solvents	Solvent-free process
Waste Generation	High waste with hazardous disposal requirements	Moderate waste requiring standard disposal	Minimal waste
Manufacturing Complexity	Complex equipment and mandatory post-processing	Complex equipment or post-processing required	Simple equipment, no post-processing needed
Material Loss	Fluffy powders and multiple processing steps	Fluffy powders or multiple processing steps	Non-fluffy product with minimal handling steps
Processing Time	>24 h total processing time	12–24 h total processing time	<4 h total processing time
Scalability	Difficult to maintain a uniform product at a large scale	Moderate scale-up challenges	Easy scale-up with uniform conditions
Excipient Safety	Volatile organic solvents	Minimal organic solvents	Non-toxic solvent-free
Processing Pressure	Vacuum/high pressure and volatile organic solvents	Vacuum/high pressure with aqueous systems	Ambient atmospheric pressure
Equipment Cleaning	Multiple complex parts require extensive cleaning	Moderate cleaning requirements	Easy cleaning process

**Table 3:** Solubility of dapagliflozin in various types of oils.

Oil Type	Solubility (mg/g)
Oleic acid	6.91 ± 0.24
Imwitor 308	212.48 ± 2.92
Peceol	67.56 ± 2.64
Captex 355	1.71 ± 0.07
Soybean oil	0.96 ± 0.15

Data presented as mean ± SD (*n* = 3).

**Table 4:** Physical appearance and percent transmittance values for the dispersed surfactant and imwitor 308 mixtures.

Surfactant Type in the Mixture	Physical Appearance	Transmittance (%)
Cremophor EL	Clear	90.38 ± 4.89
Tween 60	Pale white	65.46 ± 4.26
Span 80	Milky	4.81 ± 0.16
TO-10v	Pale white	70.40 ± 4.40

Data presented as mean ± SD (*n* = 3).

**Table 5:** The measured response values for the prepared seventeen formulations.

Runs	Liquifying Temperature (°C)	Liquifying Time (seconds)
1	29.5	84
2	32.5	100
3	30.5	90
4	34	128
5	31.5	82
6	31.5	98
7	31.5	104
8	30.5	84
9	32.5	118
10	32	94
11	32	99
12	32	91
13	30	71
14	31.5	76
15	29.5	69
16	33	87
17	34.5	120

**Table 6:** ANOVA analysis of the measured responses for the selected models.

Response	Selected Model	Degree of Freedom	*p*-Value	Lack of Fit *p*-Value	Adjusted R^2^	Predicted R^2^
liquifying temperature (°C)	Quadratic	9	<0.0001	0.8395	0.9741	0.9541
Liquifying time (seconds)	Linear	3	0.0003	0.5079	0.6930	0.5819

**Table 7:** ANOVA summary showing the estimated *p*-values for independent variables on measured responses (liquefying temperature and liquefying time).

Independent Factors	Liquefying Temperature	Liquifying Time
Pluronic F127 concentration	<0.0001	0.0025
Propylene glycol concentration	<0.0001	0.0039
Dapagliflozin concentration	0.0023	0.0033

**Table 8:** Predicted and Observed Values for the measured responses of optimized T-SNEDDS.

Analysis	Predicted Mean	Std Dev	*n*	SE Pred	95% PI Low	Data Mean	95% PI High
Liquefying temperature	33.4	0.227565	3	0.245499	32.8335	33.5	33.9945
Liquefying time	98.9	9.27142	3	7.5668	82.5866	100.3	115.281

**Table 9:** Comparative green pharmaceutical evaluation of solidification approaches.

Parameter	Lyophilization	Spray Drying	Fluid Bed Coating	Hot Melt Extrusion	Adsorption	T-SNEDDS
Environmental impact						
Energy processing	1	2	2	3	3	3
Solvent emissions	2	1	2	3	3	3
Waste streams	2	1	2	3	3	3
Cost effectiveness						
Manufacturing complexity	1	1	2	2	3	3
Materials loss	1	1	2	1	2	3
Processing time	1	2	2	2	3	3
Scalability	1	3	2	3	1	3
Operator safety						
Excipient safety	2	1	3	3	3	3
Processing pressure	2	1	2	3	3	3
Washing process	2	1	1	2	3	3

Each parameter was evaluated using a three-tier color-coded scoring system: 1 (pink, high impact/least favorable), 2 (yellow, moderate impact), and 3 (green, low impact/most favorable). Higher scores indicate better performance of the approach used across the evaluated parameters.

## Data Availability

Data are available in the manuscript.
